# The year of the nurse and midwife 2020: activating the potential and power of nursing

**DOI:** 10.1590/1518-8345.0000-3279

**Published:** 2020-06-01

**Authors:** Nancy R. Reynolds

**Affiliations:** 1 Johns Hopkins University, School of Nursing, Baltimore, MD, United States of America.

**Figure:**
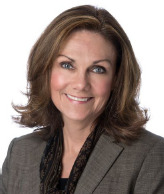


2020 is the “Year of the Nurse and Midwife.” It was designated as such by the World Health Assembly, the governing body of the World Health Organization (WHO), to recognize the work of nurses and midwives around the world and to advocate for increased investment in this workforce and for improvements in working conditions, education and professional development^(^ 1 ^)^. The year is the bicentenary of the birth of Florence Nightingale who is credited with professionalising nursing roles for women and laying the foundations for nursing of today with her groundbreaking use of statistics and the way she challenged medical dominance. Her work is also deeply imbedded in modern nursing in her recognition of the importance of lobbying and campaigning for the profession^(^ 2 ^)^.

Today, nurses are frontline workers, the largest group of health professionals in the world^(^ 2 ^)^. They play a vital role in providing essential health services at all levels of care and are crucial to promoting health and preventing disease. They care for mothers, children and the elderly, administer life-saving vaccines, and provide health advice among other actions and are often, the first and only point of care in their communities. Nurses have educational preparation that is particularly well suited to the growing challenges of the 21^st^ century characterized by a growing elder population, non-communicable diseases, multi-morbidity, fragmented health care systems, and, as seen widely in the coronavirus pandemic, critical disaster response. Nurses’ educational preparation emphasizes an integrated consideration of the patient’s physical and psychosocial health care needs. Nurses’ values are person-centered and humanitarian and they are oft regarded as the most ethical and trusted profession. They have repeatedly demonstrated competence to deliver safe and high quality patient care.

Yet, despite its inordinate value, nursing is historically plagued by gender-related oppression, lack of recognition and a lack of investment in resources to enhance educational preparation, improve workplace conditions and pay. There is an estimated shortage of over 12 million nurses. This is posed to change. Several key factors are positioned to propel the advancement of nursing this year and beyond.

1. *Dr. Tedros Adhanom Ghebreyesus, Director General of the World Health Organization*. “Dr. Tedros” is an avid proponent of the nursing profession. In his crucial leadership role for the health of the world, he repeatedly highlights the value and potential of nurses in the achievement of Universal Health Coverage and better health for all: “Nurses play a critical role not only in delivering healthcare to millions around the world, but also in transforming health policies, promoting health in communities, and supporting patients and families. Nurses are central to achieving Universal Health Coverage and the Sustainable Development Goals^(^ 3 ^)^”.
2. *Elizabeth Iro, Chief Nursing Officer at the World Health Organization (WHO)*. Elizabeth Iro is another critical asset. She has had long and esteemed career as a leader in health care^(^ 2 ^-^ 3 ^)^. Before joining WHO, Ms Elizabeth Iro had more than 30 years of experience in leadership roles in the Cook Islands and regionally. Ms. Iro now brings her substantial leadership acumen to the WHO where she is working tirelessly to advance the profile of nursing within the organization and around the globe. Among other activities she convenes the WHO Task Force on Nursing and Midwifery which provides an interdisciplinary platform to strengthen nursing leadership, advocate for political commitment, develop research and evidence, improve coordination, and embed nursing and midwifery perspectives in WHO’s work and global health initiatives. The task force is working on improving access to WHO technical guidelines and resources relevant to mainstreaming the contributions of nurses and midwives to ensure that their perspectives are made visible and explicitly addressed across strategy, policy, and programming at WHO. This is particularly important as WHO plans details on the implementation of its 13th Global Programme of Work 2019-2023-to ensure that 1 billion more people benefit from UHC, 1 billion more are protected from health emergencies, and 1 billion improve their overall health.
3. *Nursing Now Global Campaign*. Nursing Now is a three-year global campaign (2018-2020) that will culminate in 2020. The campaign was initiated to improve health by raising the profile and status of nursing worldwide. Run in collaboration with the WHO and the International Council of Nurses, Nursing Now seeks to empower nurses to take their place at the heart of tackling 21st Century health challenges and maximize their contribution to achieving Universal Health Coverage. The Nursing Now campaign is based on a 2016 report, The Triple Impact of Nursing-how developing nursing will improve health, promote gender equality and support economic growth, published by the UK All-Party Parliamentary Group on Global Health^(^ 4 ^)^ following its review of global nursing. The report concluded that UHC will not be achieved without global development of nursing. Nurses are the largest part of the professional health workforce and provide an enormous amount of care and treatment worldwide. Development of nursing will not only improve health, it will build a stronger economy and promote greater gender equity. Lord Nigel Crisp, head of the Nursing Now global campaign asserts that: “developing nursing is one of the single biggest things we can do to improve health globally.”
4. *Nursing professional organizations and scholarship*. Nursing professional organizations are making inroads to facilitate the influence of nurses in health care leadership and policy. The International Council of Nurses has, for example, been an ardent advocate for inclusion of nurses on boards and provides strong representation of nursing at the World Health Assembly (WHA). Each year, the International Council of Nurses supports a delegation who intervene on a wide range of vital issues, raising the importance of nursing’s contribution to healthcare and bringing detailed nursing advice to the highest level of policymaking in the world. Likewise, we are seeing greater influence of nurses through conduct of research and scholarship that advances the quality of care and informs policy across the globe.
5. *State of the Worlds Nursing Report*. In addition to being the Year of the Nurse and Midwife, 2020 will see the release of the first State of World Nursing Report 2020, the development of which is being led by WHO in partnership with the International Council of Nurses and the Nursing Now campaign^(^ 5 ^)^. The report will be launched on World Health Day on April 7, 2002 prior to the 73^rd^ World Health Assembly. The report will describe how the nursing workforce will help deliver Universal Health Coverage (UHC) and the Sustainable Development Goals (SDGs), and highlight areas for policy development for the next three to five years. It will also provide a technical description of the nursing workforce in Member States, including the number and types of nurses, education, regulation, practice, leadership, and gender issues. It is hoped the report will inform national policy dialogue and support decision-making on how to optimize the contributions of nurses towards PHC and UHC, unlock investment in nursing, and the gender equity agenda and accelerate progress across the SDGs.

This is an important time for nursing around the globe. These synergistic activities, achievements and recognition are indeed something for the profession to celebrate. But after we take some well-deserved time to acknowledge the unprecedented recognition during the Year of the Nurse and Midwife, we must persevere to continue to advance the contributions and influence of the nursing profession. Ongoing leadership for change will be needed around the globe to build on progress to date and the exceptional opportunities to advance nursing education and professional development, especially advanced practice nursing roles, improve working conditions and pay, build the evidence base for practice and evidence of impact, strengthen our interdisciplinary communication and collaboration, and serve actively on advisory committees, commissions, and boards where policy decisions are made to advance health systems to improve patient care. While the nursing of today looks little like the nursing of that of our predecessor, Florence Nightingale, the need for quality care, leadership and advocacy continues. We must assure that that the voice and impact of nursing continues to reverberate long after the 2020 Year of the Nurse and Midwife comes to a close.
